# Effects of Jaques–Dalcroze eurhythmics program on postural stability in elderly women

**DOI:** 10.1038/s41598-022-11095-x

**Published:** 2022-04-30

**Authors:** Jan Adamczyk, Roman Celka, Rafał Stemplewski, Kinga Ceynowa, Janusz Maciaszek

**Affiliations:** 1 Department of Dance and Gymnastics, Faculty of Sport Sciences, Poznan University of Physical Education, 61-871 Poznań, Poland; 2 Department of Physical Activity Sciences and Health Promotion, Faculty of Sport Sciences, Poznan University of Physical Education, 61-871 Poznań, Poland; 3 Faculty of Compositions, Conducting, Theory of Music and Eurhythmics, Academy of Music in Poznań, 61-808 Poznań, Poland

**Keywords:** Geriatrics, Lifestyle modification, Quality of life

## Abstract

Decreased postural stability is observed in older adults. There is an increased risk of falls, which may lead to serious complications and death. Elderly people can maintain and even improve their postural stability through properly selected exercises. This study investigated the effect of exercise using the Emil Jaques–Dalcroze’s Eurhythmics (JDE) method on the postural stability of women over 65 years of age. The study model was based on a randomized controlled trial—parallel group design. Fifty-nine women (69.85 ± 3.29) were divided into two groups: intervention (IG, n = 26) and control (CG, n = 33). The IG subjects participated in a JDE exercise programme for 12 weeks, twice a week for 45 min each. Postural stability was determined using a test on the AMTI AccuSway Plus posturography platform, recording centre of pressure (COP) point displacement. A biofeedback model was used. Accuracy, speed and reaction time of movements were assessed. Two measurement sessions were conducted in both groups: 1 week before starting the exercise programme and immediately afterwards. After 12 weeks of exercise, subjects from IG scored significantly better in the test of accuracy (p < 0.05) and speed (p < 0.05) of movements. Additionally, for parameters determining accuracy of movements, an interaction between allocation to a group and a measurement session was shown (group × time). A 12-week exercise program using the JDE method improves the postural stability of women over 65 years of age by improving the parameters of speed and accuracy of torso movements.

## Introduction

Older people experience decreased postural stability^1,2^, which causes, among others, problems in performing Activities of Daily Living (ADL)^3,4^. This in turn has a negative impact on both the psychological and physical spheres^5^, as well as increase the risk of falls^3,6^. The risk of serious complications due to falls is high^7^.

The elderly can maintain or improve their current level of postural control through well-chosen physical exercises that influence the mechanisms regulating postural control, including improved muscle strength of the lower extremity, flexibility and reaction time^8–11^. At the same time, there is evidence of heterogeneity in the response of exercisers to regular physical activity^12^. By one hand Bierbaum et al.^13^ indicated the effectiveness of only balance exercises suggesting that strength exercises do not have the intended effect. On the other hand, Burke et al.^14^ found that balance and strength exercises have the expected effect of improving postural stability in older women.

Postural stability is the ability to control the position of the body in space in order to move while maintaining balance^15^. The process of maintaining postural stability is extremely complex, involving the visual along with the central and peripheral nervous systems and the musculoskeletal system, which responds to stimuli. The quality of these responses depends on motor skills and proprioception^16^ as well as cognitive condition^17^. Taking this into account, it seems that when choosing exercises to improve postural stability, not only the physical but also the mental sphere should be involved. However, as the research results show, even such a designed exercise programme may also fail to provide inconclusive effects. Zheng et al.^18^ showed that in older people, training using a dual-task, involving simultaneous performance of cognitive tasks during physical exercise, so-called “cognicise”—a term used by Suzuki et al.^19^—improves postural control to a higher extent than physical training alone. Meanwhile, Vaillant et al.^20^ observed no significant benefit from additional cognitive exercise. Perhaps the difference in results is due to the number of training sessions performed: in the former case the subjects participated in 24 training sessions, in the latter only in 12.

One of the cognicise exercise programmes is rhythmics according to the Emil Jaques–Dalcroze’s Eurhythmics method (JDE). This method, originally developed for children, has been gaining popularity in recent years also for the elderly, but to the best of our knowledge only a few studies involving its use have been published so far^21–25^. The results show that JDE improves coordination in older adults, mainly in gait and dynamic balance. The authors describe the JDE exercises as multitasking, forcing the exercisers to focus constantly. It has been proved that exercises that consist in performing cognitive and physical tasks simultaneously provide better results than those performed separately^26^. An additional, undoubted advantage distinguishing JDE exercises is the fact that they are performed largely to the accompaniment of the piano. Apart from the practical dimension (music sets the tempo, number of repetitions, etc.), contact with live music is extremely valuable for the participants of training—the positive influence of listening to music on a person has been proven many times. It has also been proven that adding music to exercise can (a) improve exercise capacity and increase patients’ motivation to participate in cardiac and pulmonary exercise rehabilitation programs; (b) lead to improved balance, greater ability to perform activities of daily living, and improved life satisfaction in elderly individuals; (c) enhance adherence and function of individuals suffering from neurological diseases such as Alzheimer’s and Parkinson’s; and (d) sustain these benefits if continued on a long-term basis^27^. All of this creates an interesting alternative to other popular forms of exercise for seniors. Trainings are perceived as attractive and the drop-out rate during the programme is low^22^.

Yang et al.^28^ in their systematic review conclude that there is preliminary evidence supporting the positive effects of multitasking training to improve cognitive-motor skills in older people. At the same time, they point out that in many cases, the effects of interventions that combined cognitive and physical training were comparable to those that practiced these elements separately, in terms of their effects on executive function, processing speed, attention, mood and cardiorespiratory fitness. The authors point to the need for further research in this field.

In the present study, based on the experiments described above, it was decided to evaluate the effect of an exercise programme based on a 12-week gymnastic-rhythmic exercise programme using the JDE method on the postural stability of women over 65 years of age.

## Materials and methods

A study model based on randomized controlled trial—parallel group design was used. The manuscript conforms to the CONSORT Guidelines.

### Participants

Participants were recruited through advertisements in local newspapers and on the Internet, and covered the Poznań Metropolitan Area, Poland. Eligibility criteria were community-dwelling women aged 65+. Candidates had no contraindications to participation in physical activity and gave informed consent to participate in the experiment. Participants scored at least 8 on the Abbreviated Mental Ability Test^29^. Subjects taking medications that may interfere with natural control of postural stability, users of orthopaedic equipment, patients with neurological disease (Parkinson’s or Alzheimer’s), with significant visual and/or auditory perception impairment, and subjects who undertake or have undertaken regular physical activity in the past 3 years were not eligible for the programme.

Following randomization using the computer program Statistica 13.4 (TIBCO Software Inc., Palo Alto, CA), the participants were divided into two groups: Intervention Group (IG) and Control Group (CG).

### Research protocol

The participants’ postural stability was investigated by determining their ability to control the displacement of the body’s centre of pressure (COP). For this purpose, the Balance Platform model AccuSway Plus was used, together with the Balance Trainer software. The system recorded changes in COP position in the anteroposterior (AP) and mediolateral (ML) directions. The sampling rate was set at 100 Hz. The COP is a reliable parameter for evaluating postural stability and upright balance control^30^. The results of the four COP parameters described in Table 1 were statistically analysed.

**Table 1:** Description of the characteristics analysed with abbreviations, units, and abilities under study.

Parameter	Abbr.	Unit	Ability
COP Path Length: sum of path lengths from the Subject Start Position to the intersection of the active target perimeter	COP_PL	Centimetre (cm)	Accuracy of COP displacement (excluding the time to maintain the COP within the target)
COP Area Deviation: sum of areas where the COP path deviates from the straight line that intersects the Subject Start and End Positions	COP_AD	Square centimetre (cm^2^)	Accuracy of COP displacement (including the time to maintain the COP within the target)
COP Total Time: time taken to complete the entire trial	COP_TT	Seconds (s)	Movement rate of the COP
COP Reaction Time: average reaction time to cross the perimeter of target, measured from the activation of the next target	COP_RT	Seconds (s)	Simple visual-motor reaction time

### Trial design

Two measurement sessions were held: the first (baseline) took place during the week before starting the exercise programme and the second (12-week follow-up) straight after its finish (March and June 2016 respectively). Women from IG participated in the exercise programme, while women from CG were advised not to change anything about their current lifestyle, and in particular not to undertake any new structured physical activity. The participants, the JDE specialist as well as those conducting the measurements were unaware of the purpose of this study and the fact of belonging to any group.

The research project as well as all the experiments were positively assessed and approved by the Bioethics Committee at the Poznan University of Medical Sciences (Resolution 1046/15).

The trial has been registered on 20/08/2021 at ClinicalTrials.gov (Identifier: NCT05015777).

### Training programme

Training sessions took place twice a week for 45 min each over a period of 12 weeks and were held in the university gymnasium. Each training included rhythmic exercises using the Jaques–Dalcroze Eurhythmics method with piano accompaniment and electronically played music. Training sessions were conducted by a JDE specialist.

Each class consisted of three parts: warm-up (~ 10 min), main part (~ 30 min) and cool down (~ 5 min). Exercises with the use of the JDE method consist mainly in the repetition of preset musical sequences using body movements. In addition to the physical layer, the cognitive layer was equally important: while performing a given movement exercise, participants had to focus in order to react appropriately to additional tasks, such as changes in sound pitch of the piece (e.g. while pitch was high they had to walk on their toes, when low—in a half squat) as well as changes of dynamic, agogic and articulatory sequences in music. Exercises included movements based on rhythmic themes where participants had to adjust the speed of their movement to the tempo of the music, rhythmic transformation of themes and polyrhythms (the arms move in a different rhythm than the legs). There were also inhibition-incitation exercises (inhibition and stimulation of movement, i.e. stopping moving when the music stops and resuming movement when the accompaniment starts again), improvisation of movements and exercises developing control of body balance, independence of movements and their coordination^25^.

### Balance platform test

**Figure 1:**
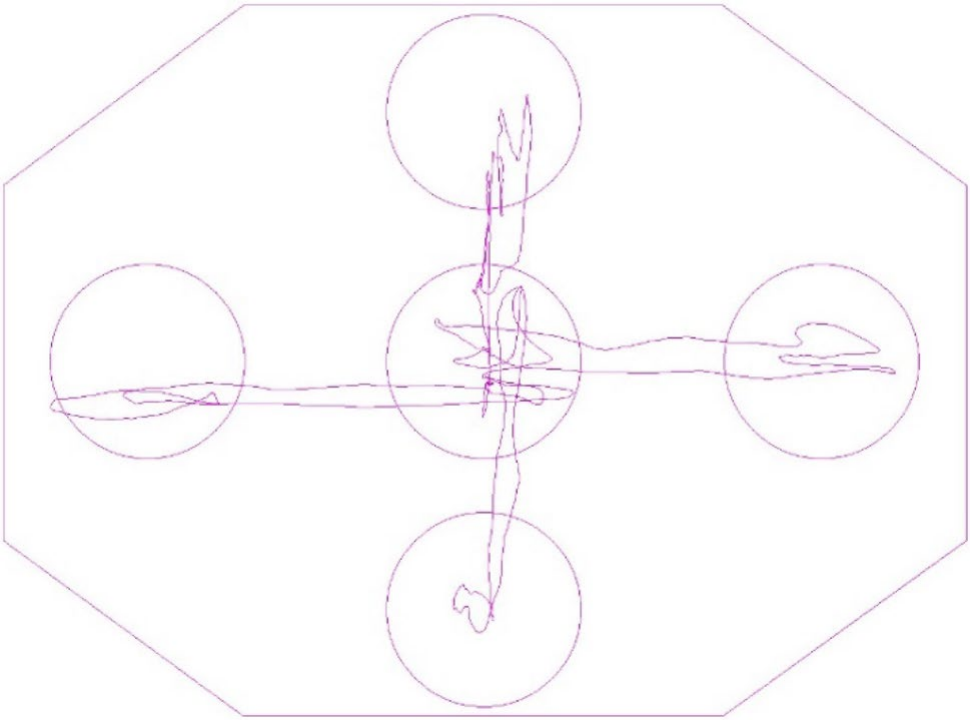
Diagram of the targets deployment with an example of the COP path trace (a screenshot from the software used for the tests).

Participants had to perform a movement task while standing on a posturographic platform. A Feedback Balance Analysis protocol was used—the subjects saw a point on the screen that was a reflection of their COP. By tilting their body in different directions, they could observe changes in the position of their COP in real time. The participants’ task was to do it in such a way as to direct their COP into the appropriate targets marked on the screen, in the right order (Fig. 1).

Taking into account the natural heterogeneity of the group in terms of physical fitness due to the age of subjects, the distances of extreme areas in relation to the central area, and thus the range of deflection, were decided to be determined for each participant individually. For this purpose, first the maximum deflections of each subject in the AP and ML directions were determined, and then the software automatically determined the centres of targets at a distance of 75% of the maximum deflection in a given direction. This ensured that each subject leaned their torso relatively the same range and that the task was within their capabilities. The results of maximum torso lean ranges were also statistically analysed.

The test consisted of two main elements: (1) the COP point movement phase and (2) the COP point maintain phase. The task consisted of the COP point movement into individual targets (in a predetermined order), each time the COP point had to be maintained inside the area of each target for at least one second, after which the target became inactive (darker), which was a signal of its correct passing. At the same time, the next (active) target would light up, indicating that the COP point had to be moved into that target, where it again had to be held for a minimum of one second, and so on. Each time the extreme target was passed, it was necessary to return into the central target. A similar trial measuring changes in postural stability in a biofeedback model under the influence of training has already been used in other experiments^31,32^.

The posturographic platform was fixed to a flat surface, in front of the subject at a distance of 1.5 m at eye level was placed a 27 inch monitor. The room was soundproofed to ensure peace and quiet. On the platform there were lines specifying the proper distances and angles of feet placement in order to ensure that the subject stood in the same place during each subsequent trial. The feet on the platform were placed naturally, one next to the other at hip width, with the toes gently pointing outwards.

Each participant was given verbal instructions on how to perform the test, with particular emphasis on the fact that the test had to be carried out as quickly and accurately as possible, moving the COP point by the shortest possible route to the target, and once reached, trying to keep the COP point as still as possible in the centre of target until it became inactive (turned dark). The person conducting the test next performed a demonstration of the entire trial. The final step was for the subjects to perform two pre-test trials, followed by a third trial, the results of which were statistically analysed.

### Statistical analysis

The results of descriptive statistics were presented as means with standard deviation (mean ± SD). Postural stability test results were presented as means and confidence intervals at the 95% level. A two-factor analysis of variance (ANOVA) was conducted. An eta square analysis was used to determine effect size. The post-hoc Tukey test was conducted in case of significant main or interaction effects. Differences between groups in the single study session area and between study sessions in the group area were determined using the Mann–Whitney *U* test with correction for continuity. Statistical significance level was set at 5%. All calculations were carried out using Statistica 13.4 (TIBCO Software Inc., Palo Alto, CA)^25^.

### Ethical approval

All procedures performed in studies involving human participants were in accordance with the ethical standards of the institutional and/or national research committee and with the 1964 Helsinki Declaration and its later amendments or comparable ethical standards.

### Informed consent

Informed consent was obtained from all subjects involved in the study.

## Results

**Figure 2:**
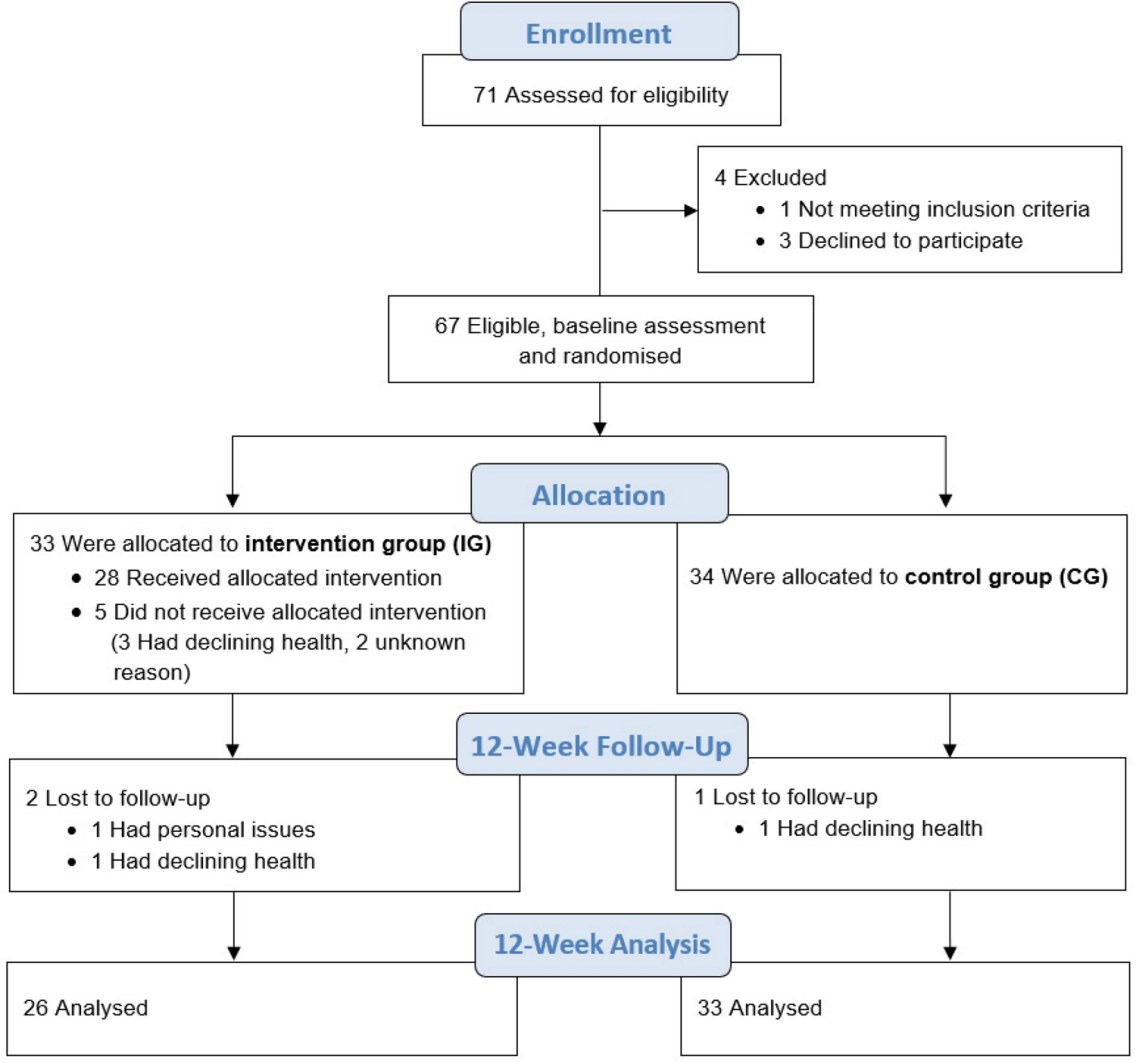
Flowchart for enrolment, randomization, and follow-up of study participants.

**Figure 3:**
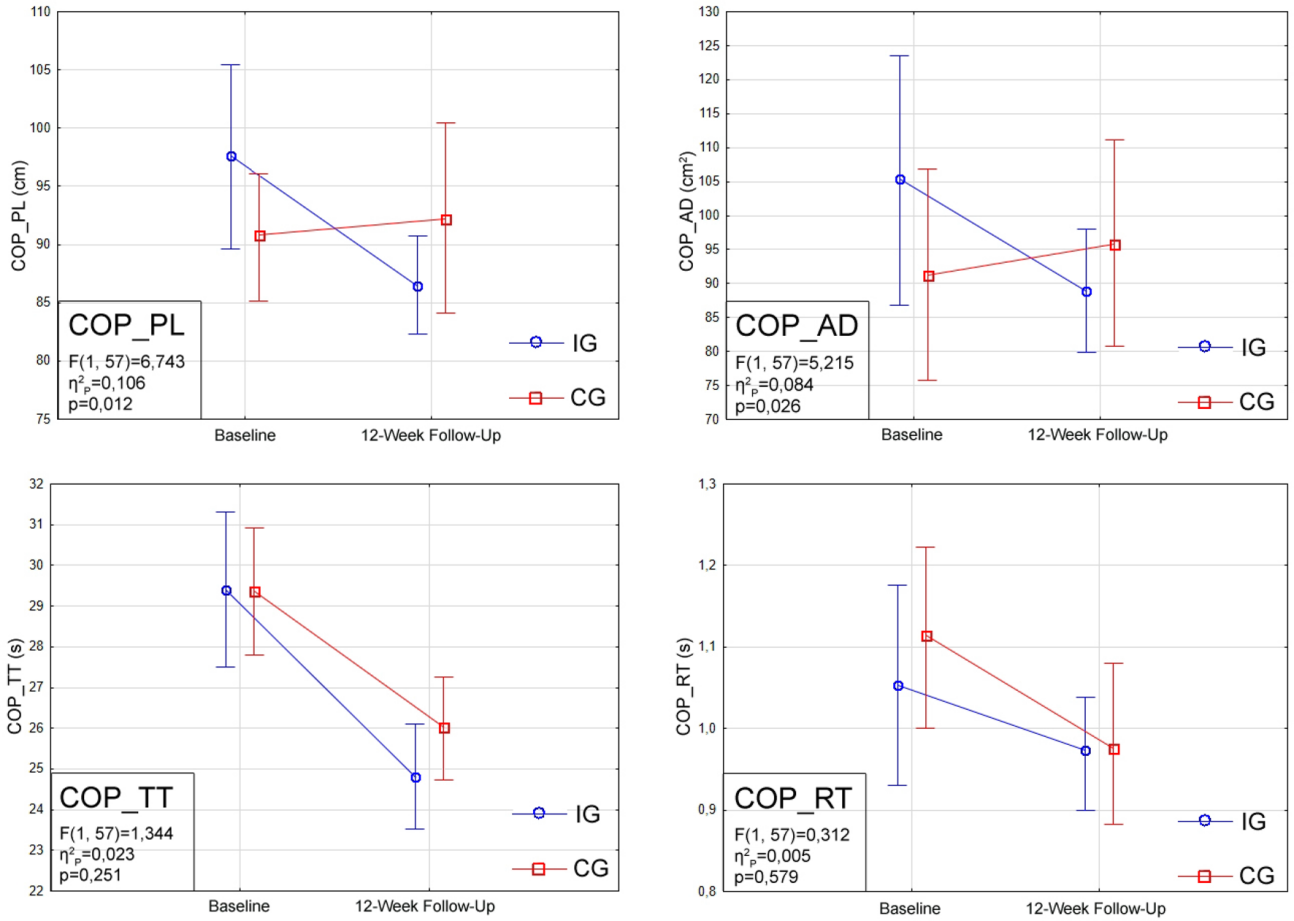
Pre- and postintervention static balance results for Centre of Pressure Path Length (COP_PL), Centre of Pressure Deviation Area Deviation (COP_AD), Centre of Pressure Total Time (COP_TT) and Centre of Pressure Reaction Time (COP_RT).

A total of 71 subjects were initially accepted for the programme, of whom 67 were qualified for the study. The final statistical analysis included a total of 59 women (Fig. 2). Table 2 presents characteristics describing the participants, including their age and the results of measurements of somatic characteristics. No significant differences were observed between IG and CG in any of the traits studied. Table 3 shows the results of postural stability tests from both groups at both times in the form of means along with 95% confidence intervals. There were no significant differences between the IG and CG groups at the baseline in any of the parameters tested. A statistically significant interaction between group allocation and measurement session (group × time interaction) was found for the parameters COP_PL (F_(1.57)_ = 6.743, p = 0.012, η^2^_P_ = 0.106) and COP_AD (F_(1.57)_ = 5.215, p = 0.026, η^2^_P_ = 0.084). Post-hoc tests of the COP_PL parameter showed significant changes (p = 0.016) for IG between the first and second measurement sessions. Figure 3 shows graphs of the direction of change in the analysed parameters along with the strength of effects and significance.

**Table 2:** Descriptive characteristics of the female participants, whose results were statistically analysed.

	ALL	IG	CG
Participants, *n*	59	26	33
Age, ${\overline{{\text{x}}}}$ (SD), years	69.85 (3.29)	69.58 (3.05)	70.06 (3.51)
Height, ${\overline{{\text{x}}}}$ (SD), cm	159.93 (5.48)	159.04 (5.05)	160.6 (5.78)
Weight, ${\overline{{\text{x}}}}$ (SD), kg	73.3 (12.41)	72.57 (12.37)	73.28 (12.68)
BMI, ${\overline{{\text{x}}}}$ (SD), kg/m^2^	28.73 (5.08)	28.74 (5.00)	28.47 (5.22)
**Education level**, n (%)			
Secondary	31 (52.5)	10 (38.5)	21 (63.6)
Higher	17 (28.8)	11 (42.3)	6 (18.8)
Vocational	6 (10.2)	3 (11.5)	3 (9.1)
PhD	4 (6.8)	2 (7.7)	2 (6.1)
Primary	1 (1.7)	0 (0)	1 (3)
**Marital status**, n (%)			
Married	25 (42.4)	12 (46.2)	13 (39.4)
Widow	22 (37.3)	8 (30.7)	14 (42.4)
Single	5 (8.5)	1 (3.9)	4 (12.2)
Divorcée	4 (6.8)	3 (11.5)	1 (3)
Separation	3 (5.1)	2 (7.7)	1 (3)
**Weekly PA frequency**, n (%)			
3–4 ×/week	17 (28.8)	7 (26.9)	10 (30.3)
Every day	16 (27.1)	8 (30.8)	8 (24.2)
None	12 (20.3)	6 (23.1)	6 (18.2)
1–2 ×/week	8 (13.6)	3 (11.5)	5 (15.2)
5–6 ×/week	6 (10.2)	2 (7.7)	4 (12.1)

*IG* Intervention Group, *CG* Control Group, *n* number of observations, $\overline{\mathrm{x}}$ mean, *SD* standard deviation, *PA* physical activity.

**Table 3:** Descriptive data—means and 95% confidence interval (CI) of COP_PL (cm), COP_AD (cm^2^), COP_TT (s) and COP_RT (s) at the baseline and at the 12-week follow-up in both intervention and control group.

	Baseline		12-Week follow-up
*COP_PL, cm			
IG	97.58 (89.67–105.50)	—*p *= *0.01*→	86.41 (82.21–90.62)
CG	90.81 (85.07–96.55)		92.21 (84.05–100.38)
*COP_AD, cm^**2**^			
IG	105.40 (86.88–123.92)		88.91 (79.81–98.01)
CG	91.25 (75.78–106.71)		95.84 (80.50–111.19)
**COP_TT, s**			
IG	29.39 (27.50–31.28)	—*p* < *0.01*→	24.79 (23.53–26.06)
CG	29.36 (27.84–30.89)	—*p* < *0.01*→	26.02 (24.75–27.29)
**COP_RT, s**			
IG	1.05 (0.93–1.18)		0.97 (0.9–1.04)
CG	1.11 (1.0–1.22)		0.98 (0.88–1.08)

*COP_PL* path length of the centre of pressure, *COP_AD* area deviation of the centre of pressure, *COP_TT* total time of the centre of pressure activity, *COP_RT* reaction time of the centre of pressure, *IG* Intervention Group, *CG* Control Group. Test marked with *—there was significant interaction (p < 0.05) between group and measurement session (Group × Time interaction). Differences within group between baseline and 12-week follow-up are indicated as p value.

## Discussion

The biofeedback trials conducted on the posturography platform showed that the training with the JDE method can contribute to increasing the efficiency of using vision in order to keep the body in a stable position. The overall image of torso leaning of the female subjects shows that all analysed parameters improved. Parameters determining the speed of movement of the moving COP, accuracy, and visuomotor reaction time were evaluated in detail. For the assessment of movement precision, two parameters were analysed: (1) COP_PL, which determines the accuracy of COP control during the movement phase, and (2) COP_AD, which additionally indicates the ability to maintain the body in a forced position. In both cases, a significant statistical interaction was found between group allocation and measurement session. Although the post-hoc test showed a significant change—improvement in IG performance—only in the case of COP_PL, the direction of change in the performance of both analysed parameters is opposite in the IG group compared to the CG. This means that JDE training had a positive effect on the ability to control body leaning during forced movements on the posturographic platform. Hernandez et al.^33^ observed that older women leaning their whole body (moving their COP) performed additional corrective submovements. It can be concluded here that, in the case of IG, the JDE exercises reduced their number, thus affecting the smoothness and precision of movement phase more than maintaining the COP in a forced position (inside the target on the monitor screen).

In order to study not only the correctness of movements but also their speed, the total time to perform this forced trial (COP_TT) was measured. It was assumed that the lower the score, the faster and therefore better the COP movements performed. In order to exclude misinterpretation of this result, which could arise in a situation in which the subject reacts too slowly to the target deactivation signal (significantly prolonging the maintenance phase), it was also decided to analyse the reaction time (COP_RT) which was needed to leave the target after passing it. No significant group × time interaction was found in any of the above parameters, but detailed statistical analysis showed a significant improvement in speed parameters in the 12-week Follow-Up for both groups. It is possible that, despite the fact that subjects from both groups (including CG) were not allowed to change their level of physical activity and perform balance exercises at home on their own, they performed body leaning trials. It could have affected the ability to lean the body in different directions, resulting in changes of COP_TT and COP_RT parameters, probably more easily trainable, in both groups. Additionally, there may have been a learning effect. Furthermore, perhaps these two parameters are too sensitive to the effects of any training and should only be used in combination with other posturographic parameters (e.g. COP_PL and COP_AD).

From the experiment, based on the changes in all analysed parameters, it is evident that the training with JDE resulted in an improvement in both the precision and speed of volitional movements of the COP in the test with biofeedback. The movements performed by subjects in the IG group were less extensive and thus performed with greater precision. With the slow but coordinationally challenging movements, learned and applied over a period of 12 training weeks, the whole-body control and proprioception were clearly improved. Furthermore, studies on the organization and control of human movements^33^ show that it is not only the condition of internal organs directly involved in the movements, or even the quality of nerve connections, that is important. Apart from the automatism of the body’s functioning, there are structures of the cortex of brain where the decision is made—whether or not to perform a movement despite sufficient data from the receptors. Information from the receptors is constantly flowing into the brain, but making the right response is a matter of choice, and the decision is made in the appropriate centres^34^. It is likely that the exercises, often complex in terms of coordination, resulted in an increase in confidence in the control of movements of the whole body and enabled faster and better choices of the correct, accurate movement. The JDE training method is similar to Tai-Chi and dance in its use of coordinative elements, which also have a significant influence on the control of movements—thus leading to greater postural stability^35,36^ The JDE also requires adequate control of individual muscle activity. Hence, in women undergoing JDE training, an improvement in performance was noted in the standing test on the posturographic platform with feedback. This improvement should be associated with an increased ability to use visual stimuli in order to gather information necessary to hold the body in the desired position. The torso leaning of older women aimed at keeping the body upright on the platform in such a way that the COP was in one specific position became more controlled and precise after 12 weeks of training.

As the exercise program was based on the use of music, its influence on the results should also be considered. Hagen et al.^37^ found that a group of seniors who exercised with music, compared to a control group that exercised without music, had better results in the areas of balance, joint range of motion, cognitive ability, behavioural assessment and life satisfaction. Similar positive results were obtained by Hamburg and Clair^38^—the exercise program consisted of a selection of music that matched the rhythm of exercises being performed. After 5 weeks of exercise, seniors reported significant improvements in balance, flexibility, coordination, and spatial orientation. The study also points to other positive changes in exercise with music besides motor skills that can be achieved by seniors. Teel et al.^39^ reported an extremely important high participation rate in such activities (> 89%) in this group of people. In addition, self-reported high motivation to exercise, increased postural awareness, improved balance and increased social interaction were reported by the participants. Meanwhile, Carrick et al.^40^ in their randomized controlled trail show that even just listening to music can improve postural stability and reduce the risk of falls. All of the above characteristics may have contributed to better outcomes for the IG participants in this study.

This randomized, long-term experiment based on the JDE method obviously has some limitations: (1) In order to draw conclusions about changes in brain structures due to JDE training, it would be necessary to determine in which brain regions there was an increase in activity. For this purpose functional Magnetic Resonance Imaging (fMRI) should be used, which was not the case in this study. (2) It should be noted that the model of study consisting of individual determination of the distance of extreme targets from the centre makes it impossible to compare the subjects’ results with each other (and thus to define standards) due to the different distances that the COP point has to cover. However, the creation of such standards would be of little scientific or practical interest. To create such comparisons, it would be necessary to adopt a qualifying criterion for subjects to achieve a given minimum of torso lean in each of the four directions and then set the end of extreme targets at that distance—but this, in turn, would significantly reduce the standardization of this type of measurement. (3) The lack of calculation of sample size. In determining the minimum number of participants we relied on other similar studies. It is advisable that for subsequent studies, the number of participants should be determined based on the pilot study conducted earlier. (4) In designing the study, we decided to use only the platform test as the outcome measure. We assumed that the biofeedback protocol assesses changes in the physical and cognitive spheres simultaneously, i.e. those affected by the JDE exercises. However, in order to obtain more comprehensive results, additional tests such as the Mini Balance Evaluation System Test, Timed Up and Go, One Leg Stand as well as balance and falls confident skills tests should be used in future studies. (5) Comparing the intervention group only with a control group whose participants do not participate in any activities. To better control for the strength of social interaction effects, future studies should include not only the control group as here, but also a social intervention group such as a book or cards club or other type of social gathering that does not involve physical activity or exercise. (6) Participants should have had an extra day before baseline, dedicated to familiarisation process on the platform, to educate them and have them practise before the actual baseline test day.

## Conclusions

A 12-week exercise programme using the JDE method improves postural stability in the dynamics of women over 65 years of age, by improving the smoothness of torso movements during body leaning similar to those occurring in everyday life, thereby improving the performance of ADLs and reducing the risk of falls. This form of exercise is safe, effective and attractive for seniors. Due to its nature (dance, music, rhythm, whole body movements) this form can be used in activating and improving people who do not like traditional gymnastics on a daily basis. The obtained results may be particularly interesting for people conducting physical activities in nursing homes and centres for the elderly, where the proposed forms of therapy must be characterised by high attractiveness and ease of evoking positive emotions.

In the light of the small number of publications to date that have examined the effects of JDE on older adults, further research is needed to better understand the mechanism by which these exercises affect functional fitness in elderly people.

## Data Availability

The data presented in this study are available on reasonable request from the corresponding author.

## References

[1] Leightley D, Yap M, Coulson J, Piasecki M, Cameron J, Barnouin Y, Tobias J, McPhee J (2017). Postural stability during standing balance and sit-to-stand in master athlete runners compared with nonathletic old and young adults. J. Aging Phys. Act..

[2] Alsubaie SF (2020). The postural stability measures most related to aging, physical performance, and cognitive function in healthy adults. BioMed Res. Int..

[3] Bukova A, Hagovska M, Drackova D, Horbacz A, Wasik J, Krucanica L (2019). Awareness of patients suffering from selected chronic diseases of the importance of physical activity in treating their disorders. Phys. Act. Rev..

[4] Sabashi K, Ishida T, Matsumoto H, Mikami K, Chiba T, Yamanaka M, Aoki Y, Tohyama H (2021). Dynamic postural control correlates with activities of daily living and quality of life in patients with knee osteoarthritis. BMC Musculoskelet. Disord..

[5] Miyawaki T, Kumamoto K, Shimoda K, Tozato F, Iwaya T (2017). Relationship among motor function, ADL disability, and psychological concerns in elderly people with locomotive disorders. J. Orthop. Sci..

[6] Hamed A, Bohm S, Mersmann F, Arampatzis A (2018). Exercises of dynamic stability under unstable conditions increase muscle strength and balance ability in the elderly. Scand. J. Med. Sci. Sports.

[7] Alamgir H, Muazzam S, Nasrullah M (2012). Unintentional falls mortality among elderly in the United States: Time for action. Injury.

[8] Taheri M, Irandoust K, Moddaberi S (2019). The effects of weight-bearing exercise on postural control and fatigue index of elderly males. Int. Arch. Health Sci..

[9] Alfieri F, Riberto M, Abril-Carreres A, Boldó-Alcaine M, Rusca-Castellet E, Garreta-Figuera R, Battistella L (2012). Effectiveness of an exercise program on postural control in frail older adults. Clin. Interv. Aging..

[10] Stemplewski R, Maciaszek J, Salamon A, Tomczak M, Osinski W (2012). Effect of moderate physical exercise on postural control among 65–74 years old men. Arch. Gerontol. Geriatr..

[11] Judge J, Lindsey C, Underwood M, Winsemius D (1993). Balance improvements in older women: Effects of exercise training. Phys. Ther..

[12] Bouchard C, Rankinen T (2001). Individual differences in response to regular physical activity. Med. Sci. Sports Exerc..

[13] Bierbaum S, Peper A, Arampatzis A (2013). Exercise of mechanisms of dynamic stability improves the stability state after an unexpected gait perturbation in elderly. Age (Dordr.).

[14] Burke TN, Franca FJR, de Meneses SRF, Cardoso VI, Marques AP (2020). Postural control in elderly persons with osteoporosis: Efficacy of an intervention program to improve balance and muscle strength. A randomized controlled trial. Am. J. Phys. Med. Rehabil..

[15] Woollacott M, Shumway-Cook A (2002). Attention and the control of posture and gait: A review of an emerging area of research. Gait Posture.

[16] Goudarzian, M., Rahimi, M., Karimi, N., Samadi, A., Ajudani, R., Sahaf, R., & Ghavi, S. Mobility, balance, and muscle strength adaptations to short-term whole body vibration training plus oral creatine supplementation in elderly women [Research Article]. Asian J. Sports Med. 8(1). 10.5812/asjsm.36793 (2017).

[17] Cieślik B, Chamela-Bilińska D, Ostrowska B, Szczepańska-Gieracha J (2019). Postural instability in cognitively impaired elderly during forward and backward body leans. J. Phys. Ther. Sci..

[18] Zheng J, Pan Y, Hua Y, Shen H, Wang X, Zhang Y, Fan Y, Yu Z (2013). Strategic targeted exercise for preventing falls in elderly people. J. Int. Med. Res..

[19] Suzuki T, Makizako H, Doi T, Park H, Lee S, Tsutsumimoto K, Umemura K, Maki Y, Shimada H (2015). Community-based intervention for prevention of dementia in Japan. J. Prev. Alzheimer's Dis..

[20] Vaillant J, Vuillerme N, Martigné P, Caillat-Miousse J, Parisot J, Nougier V, Juvin R (2006). Balance, aging, and osteoporosis: Effects of cognitive exercises combined with physiotherapy. Joint Bone Spine.

[21] Kressig R, Allali G, Beauchet O (2005). Long-term practice of Jaques Dalcroze eurythmics prevents age-related increase of gait variability under dual task. J. Am. Geriatr. Soc..

[22] Trombetti A, Hars M, Herrmann FR, Kressig RW, Ferrari S, Rizzoli R (2011). Effect of music-based multitask training on gait, balance, and fall risk in elderly people: A randomized controlled trial. Arch. Intern. Med..

[23] Hars M, Herrmann FR, Fielding RA, Reid KF, Rizzoli R, Trombetti A (2014). Long-term exercise in older adults: 4-year outcomes of music-based multitask training. Calcif. Tissue Int..

[24] Ferguson-Stegall L, Vang M, Wolfe AS, Thomsen KM (2017). A 9-week Jaques–Dalcroze eurhythmics intervention improves single and dual-task gait speed in community-dwelling older people. J. Phys. Act. Health.

[25] Adamczyk J, Celka R, Stemplewski R, Ceynowa K, Kamińska P, Maciaszek J (2020). The impact of 12-week Jaques–Dalcroze eurhythmics programme on the dynamic agility in single-dual-task conditions in older women: A randomized controlled trial. BioMed Res. Int..

[26] Tait J, Duckham R, Milte C, Main L, Daly R (2017). Influence of sequential vs. simultaneous dual-task exercise training on cognitive function in older adults. Front. Aging Neurosci..

[27] Ziv G, Lidor R (2011). Music, exercise performance, and adherence in clinical populations and in the elderly: A review. J. Clin. Sport Psychol..

[28] Yang C, Moore A, Mpofu E, Dorstyn D, Li Q, Yin C (2020). Effectiveness of combined cognitive and physical interventions to enhance functioning in older adults with mild cognitive impairment: A systematic review of randomized controlled trials. Gerontologist.

[29] Jitapunkul S, Pillay I, Ebrahim S (1991). The abbreviated mental test: Its use and validity. Age Ageing.

[30] Chien J-E, Hsu W-L (2018). Effects of dynamic perturbation-based training on balance control of community-dwelling older adults. Sci. Rep..

[31] Maciaszek J, Borawska S, Wojcikiewicz J (2014). Influence of posturographic platform biofeedback training on the dynamic balance of adult stroke patients. J. Stroke Cerebrovasc. Dis..

[32] Maciaszek J, Osiński W, Szeklicki R (2006). Age, BMI, psychomotor and functional fitness as determinants of static and dynamic balance in elderly men with osteopenia or osteoporosis. Stud. Phys. Cult. Tour..

[33] Hernandez M, Ashton-Miller J, Alexander N (2012). The effect of age, movement direction, and target size on the maximum speed of targeted COP movements in healthy women. Hum. Mov. Sci..

[34] Brass M, Haggard P (2007). To do or not to do: The neural signature of self-control. J. Neurosci..

[35] Xu D, Li J, Hong Y (2003). Tai Chi movement and proprioceptive training: A kinematics and EMG analysis. Res. Sports Med..

[36] Miura A, Fujii S, Yamamoto Y, Kudo K (2015). Motor control of rhythmic dance from a dynamical systems perspective: A review. J. Dance Med. Sci..

[37] Hagen B, Armstrong-Esther C, Sandilands M (2003). On a happier note: Validation of musical exercise for older persons in long-term care settings. Int. J. Nurs. Stud..

[38] Hamburg J, Clair A (2003). The effects of a movement with music program on measures of balance and gait speed in healthy older adults. J. Music Ther..

[39] Teel C, Carson P, Hamburg J, Clair AA (1999). Developing a movement program with music for older adults. J. Aging Phys. Act..

[40] Carrick F, Oggero E, Pagnacco G (2007). Posturographic changes associated with music listening. J. Altern. Complement. Med..

